# Markers of humoral and cell-mediated immune response in primary autoimmune hypophysitis: a pilot study

**DOI:** 10.1007/s12020-021-02612-5

**Published:** 2021-01-23

**Authors:** Sabrina Chiloiro, Antonella Giampietro, Flavia Angelini, Vincenzo Arena, Egidio Stigliano, Tommaso Tartaglione, Pier Paolo Mattogno, Quintino Giorgio D’Alessandris, Liverana Lauretti, Alfredo Pontecorvi, Laura De Marinis, Antonio Bianchi

**Affiliations:** 1 Pituitary Unit, Department of Endocrinology and Diabetes, Fondazione Policlinico Universitario A. Gemelli, IRCCS, Rome, Italy; 2 Dipartimento di Medicina e Chirurgia traslazionale, Università Cattolica del Sacro Cuore, Rome, Italy; 3 Laboratory of Vascular Biology and Genetics, Department of Translational Medicine and Surgery, Università Cattolica del Sacro Cuore, Rome, Italy; 4 Department di Pathology, Fondazione Policlinico Universitario A. Gemelli, IRCCS, Rome, Italy; 5 Istitute of Pathology, Università Cattolica del Sacro Cuore, Rome, Italy; 6 Radiology Unit, Istituto Dermopatico dell’Immacolata-IRCCS-FLMM, Rome, Italy; 7 Department of Neurosurgery, Fondazione Policlinico Universitario A. Gemelli, IRCCS, Università Cattolica del Sacro Cuore, Rome, Italy

**Keywords:** Hypopituitarism, Autoimmunity, Anti-pituitary antibody, Cell mediated immune response

## Abstract

**Introduction:**

Primary autoimmune hypophysitis (PAHs) is a rare inflammatory disease of the pituitary gland. Although largely investigated, the pathogenesis of PAH is not completely clarified. We aimed to investigate the immune response in PAHs.

**Material and methods:**

Serum anti-pituitary and anti-hypothalamus antibodies (respectively APAs and AHAs) were investigated though an indirect immunofluorescence on monkey hypophysis and hypothalamus slides, serum cytokines though an array membrane and cell-mediated immunity though the white blood cells count.

**Results:**

Nineteen PAH cases entered the study. APA or AHA were identified in all cases. APA were detected in 13 patients (68.4%) and AHA in 13 patients (68.4%). Ten patients (52.6%) were simultaneously positive for both APA and AHA. The prevalence of APAs and AHAs was higher as compared to those observed in 50 health controls (respectively 14% *p* < 0.001 and 24% *p* = 0.004) and in 100 not-secreting pituitary adenoma (NFPAs) (respectively 22% *p* = 0.002 and 8% *p* < 0.001). Similarly, the prevalence of simultaneous positivity for APA and AHA (52.9%) was higher as compared to the those detected in patients affected by NFPAs (0%; *p* < 0.001) and in health controls (16% *p* = 0.002). No differences were identified between PAHs and controls at qualitative and quantitative analysis of serum cytokines and white blood cells count.

**Conclusions:**

This study suggest that APA and AHA may be detected in an high percentage of PAH cases and that their simultaneous identification may be useful for the differential diagnosis between PAH and NFPAs, in an appropriate clinical context.

## Introduction

Primary autoimmune hypophysitis (PAHs) is a rare inflammatory disease of the pituitary gland and represents an emerging problem, as in recent years, an increased number of cases has been described [1].

The pituitary gland is high susceptible to autoimmune damage, being a highly vascularized peripheral organ outside the blood brain barrier. Moreover, a large amount of proteins may act as antigens, as hormones, pre-hormones and hypothalamic releasing hormones, that are storage and synthetized in the pituitary gland. In addition, the endothelial cells lining the pituitary sinusoids provide a little barrier to the passage of secreted proteins from the endocrine cells to the bloodstream, which drain rapidly via the cavernous sinus to the jugular veins. In the most recent years, a possible role of the meningeal compartment in regulating the immune surveillance was also described. Recent studies have described the presence of a meningeal-lymphatic network, called “glymphatic system”. Within the glymphatic system, cerebrospinal fluid enters the brain via peri-arterial spaces, passes into the interstitium via perivascular astrocytic aquaporin-4 and then drives the perivenous drainage of the interstitial fluids and its solute [2]. This recently discovered glymphatic system represents a novel pathway for the drainage of the cerebrospinal fluid and a more conventional path for the immune cells to egress the central nervous system [3, 4]. The dysfunction of the glymphatic system was described in neurological diseases, associated with protein accumulation within the central nervous system and immune disorders, as the multiple sclerosis and the Alzheimer’s disease.

The pathogenesis of PAH is not completely clarified, at the moment, although several studies were conducted to identify the putative antigens, the HLA genotypes, the cell-mediated immune response [5–14]. In this study, we tried to investigate the immune response in our cohort of PAHs patients, though the determination of serum anti-pituitary and anti-hypothalamus auto-antibodies (respectively APAs and AHAs), of serum cytokines and of cell-mediated immunity.

## Patients and methods

A monocentric study was conducted on 19 PAH cases.

Diagnosis of PAH was conducted, according to clinical criteria in most cases, as described in our previous studies [15–17]. All the following criteria have to be satisfied:

1. Occurrence of hypopituitarism and/or hyperprolactinemia and/or diabetes insipidus and/or visual field deficit and/or headache
2. Identification of the typical hypophysitis findings [18] through a pituitary magnetic resonance (MR), as pituitary enlargement, pituitary stalk swelling and absence of the posterior pituitary “bright spot” on T1-weighed (T1-w) images;
3. Exclusion of focal hypothalamic-pituitary lesions/masses;
4. Exclusion of secondary causes as granulomatous vasculitis, sarcoidosis, Langerhans cell histiocytosis and tuberculosis [19].

Histological diagnosis though pituitary biopsy was performed in selected cases as those with worsening of hypophysitis during immunosuppressive treatment and in cases with diagnosis in doubt.

The immune response was investigated though the determination of APAs and AHAs, serum cytokines and cell-mediated immunity. Blood and serum was collected at the time of the PAH diagnosis and before starting any treatment.

### Determination of APAs and AHAs

As described by several authors [15, 20, 21], APAs and AHAs were detected, by an indirect immunofluorescence method on monkey hypophysis slides (MHY) and monkey hypothalamus slides (MTH) supplied by Biosystem, S.A. (Barcelona 2010). The slides were provided with pre-absorbed IgG FITC by Biosystem, S.A. (Barcelona 2010). Serum APA and/or AHA bind to the corresponding antigens present on monkey sections. The antigen-antibody complexes are detected by means of a goat anti-human IgG conjugated with fluorescein isothiocyanate (FITC). IgG FITC was adsorbed with monkey serum to remove non-specific fluorescence. Pre-adsorption is a step whereby the secondary antibodies were exposed to immobilized monkey serum to remove non-specific antibodies from the solution. This step increases the specificity of the adsorbed antibodies, thus reducing the risk of non-specific background fluorescence. Samples were considered positive when a diffuse immunofluorescence pattern with an intracytoplasmic staining was observed in the majority of fields. In each assay, a positive and negative control was included. Sera of patients were considered positive for APA and/or AHA starting at a dilution rate of 1:8.

### Cytokine array

Human Cytokine Antibody Array (ab133997, Abcam) was used for the simultaneous detection of 42 cytokines in each serum sample according to the manufacturer’s instructions (ENA-78, GCSF, GM-CSF, GRO, alpha-GRO, I-309, alpha IL-1, beta-IL1, IL-2, IL-3; IL-4; IL-5; IL-6; IL-7; IL-8; IL-10; IL-12p40/p70; IL-13; IL-15; gamma-INF;MCP-1; MCP-2; MCP-3; MCSF; MDC; MIG; delta-MIP-1; RANTES, SCF, SDF-1; TARC; TGF-beta1; alpha-TNF; beta-TNF; EGF; IGF-I, Angiogenina, Oncostatin; Thrombopoietin, VEGF; PDGF-bb; Leptin). The array membranes were incubated for 30 min at room temperature in blocking buffer. Serum samples were then incubated on the membranes overnight at 4 °C on a rocking platform shaker. Following four washes in wash buffer I and three washes in wash buffer II, membranes were incubated in Biotin-Conjugated Anti-Cytokines for 2 h at room temperature. After washing, membranes were incubated in HRP-Conjugated Streptavidin for 2 h at room temperature. Washed arrays were finally incubated with Chemi luminescence Detection reagents and images were captured on ChemiDOC (Bio-rad). Pixel density (signal density) of each spot on membrane was quantified using Image Lab 4.0 software (Bio-rad), and corrected for background intensity.

### Cell-mediated immunity

The systemic inflammatory response was investigated by the neutrophil and lymphocytes counts and by the neutrophil/lymphocyte ratio, as described in other autoimmune diseases [22–24].

## Statistical analysis

The patients’ cohort was described in its clinical and demographic features using descriptive statistics techniques. Normality of continuous variables was checked using Kolmogorov-Smirnov test. Quantitative variables were expressed as mean and standard deviation or median and interquartile ranges, as appropriate. The qualitative variables as absolute and percentage frequency. Chi square test (or Fisher exact test when necessary), parametric and non-parametric tests were used to compare categorical and quantitative un-paired data, as appropriate. Odds ratios (OR) were calculated as calculated as measures of risk. In order to define the APA and AHA sensibility and specificity in identify cases of PAHs, the prevalence of APA and AHA in the study population was compared to those observed in a group of health controls (normal donors) and in a group of patients affected by not-secreting pituitary adenoma (NFPAs). Assuming alpha = 0.05 and power = 80% and an overall event proportion lower than 0.02 in health controls and of 0.10 in NFPAs [25], sample size was estimated to 30 cases for health controls and to 100 cases for NFPA. The analyses were performed using SPSS software version 24.0 for Windows.

## Ethical approval

All procedures performed in studies involving human participants were in accordance with the ethical standards of the institutional review board and with the 1964 Helsinki declaration and its later amendments or comparable ethical standards. The study was approved by Institutional Review Board of the Gemelli Hospital, Catholic University of the Sacred Heart, Rome. All patients entered the study signed an informed consensus

## Results

**Fig. 1:**
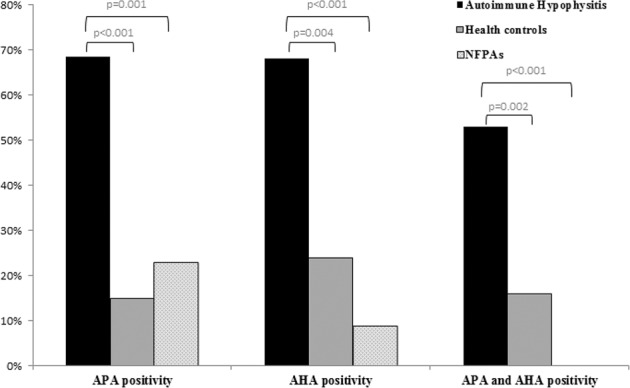
Histogram that represents the prevalence of anti-pituitary (APA) and anti-hypothalamus (AHA) antibodies in patients affected by primary autoimmune hypophysitis, in not-secreting pituitary adenoma and in heath controls

Thirteen of the 19 patients entered the study were females (68.4%). Mean age at diagnosis was 39 years (SD: 19). APA or AHA were identified in all cases: APA were detected in 13 patients (68.4% of cases) and AHA in 13 patients (68.4% of cases). Ten patients (52.6% of cases) were simultaneously positive for both APA and AHA. The positivity for APA and AHA did not differ among the different subtypes of hypophysitis and did not correlated with the occurrence of pituitary dysfunction, as shown in Table 1. In order to investigate the sensibility and specificity of APA and AHA in the diagnosis of PAH, the results of immunofluorescence of serum of patients with PAHs were compared with those of 100 patients affected by NFPAs and 50 health controls. As shown in Fig. 1, the prevalence of APA (68.4%) was higher in PAHs as compared to controls (14% *p* < 0.001, sensibility: 75%, specificity: 86%) and to NFPAs (22% *p* = 0.002; sensibility: 76%, specificity: 77%). Similarly, the prevalence of AHAs (68.4%) was higher in patients with PAHs as compared to health control (24% *p* = 0.004; sensibility: 76%, specificity: 76%) and to NFPAs (8%; *p* < 0.001, sensibility: 76%, specificity: 92%). The detection of APA and AHA was protective from the diagnosis of NFPAs with an odds ratio respectively of 0.35 (95% CI: 0.17–0.69) and 0.21 (95% CI:0.11–0.41). The prevalence of coexisting positivity for APA and AHA (52.9%) was higher as compared to the those detected in patients affected by NFPAs (0%; *p* < 0.001) and in health controls (8 of 50 cases: 16% *p* = 0.002). The detection of coexisting APA and AHA was protective from the diagnosis of NFPAs with an odds ratio of 0.27 (0.13–0.57).

**Table 1:** Clinical features of patients affected by primary autoimmune hypophysitis according to the immunofluorescence pattern of anti-pituitary and anti-hypothalamus antibodies

	Anti-pituitary antibodies			Anti-hypothalamus antibodies		
	Positivity	Negativity	*p* value	Positivity	Negativity	*p* value
Gender *n*, (%)						
Females	9 (69.2%)	4 (66.7%)	0.652	8 (61.5%)	5 (83.3%)	0.348
Males	4 (30.8%)	2 (33.3%)		5 (38.5%)	1 (16.7%)	
Mean age at AH diagnosis	41 (15)	31 (16)	0.162	39 (16)	35.1 (25)	0.594
Hypophysitis subtypes *n*, (%)						
Adeno-hypophysitis	5 (38.5%)	3 (50%)	0.44	6 (46.2%)	2 (33.3%)	0.246
Pan-hypophysitis	3 (23%)	0		3 (23.1%)	0	
Infundibulo-neuro-hypophysitis	5 (38.5%)	3 (50%)		4 (30.7%)	4 (66.7%)	
Secondary hypothyroidism *n*, (%)						
Yes	1 (7.7%)	0	0.684	1 (7.7%)	0	0.684
No	12 (92.3%)	6 (100%)		12 (92.3%)	6 (100%)	
Secondary hypogonadism *n*, (%)						
Yes	5 (38.5%)	2 (33.3%)	0.622	4 (30.8%)	3 (50%)	0.378
No	8 (61.5%)	4 (66.7%)		9 (69.2%)	3 (50%)	
Secondary hypoadrenalism *n*, (%)						
Yes	4 (30.8%)	2 (33.3%)	0.652	5 (38.5%)	1 (16.7%)	0.348
No	9 (69.2%)	4 (66.7%)		8 (61.5%)	5 (83.3%)	
Growth hormone deficit *n*, (%)						
Yes	2 (15.4%)	1 (16.7%)	0.705	1 (7.7%)	2 (33.3%)	0.222
No	11 (84.6%)	5 (83.3%)		12 (92.3%)	4 (66.7%)	
Hyperprolactinemia *n*, (%)						
Yes	3 (23.1%)	1 (16.7%)	0.627	3 (23.1%)	1 (16.7%)	0.627
No	10 (76.9%)	5 (83.3%)		10 (76.9%)	5 (83.3%)	
Diabetes insipidus *n*, (%)						
Yes	7 (53.8%)	3 (50%)	0.63	6 (46.2%)	4 (66.7%)	0.37
No	6 (46.2%)	3 (50%)		7 (53.8%)	2 (33.3%)

### Cytokine array

**Fig. 2:**
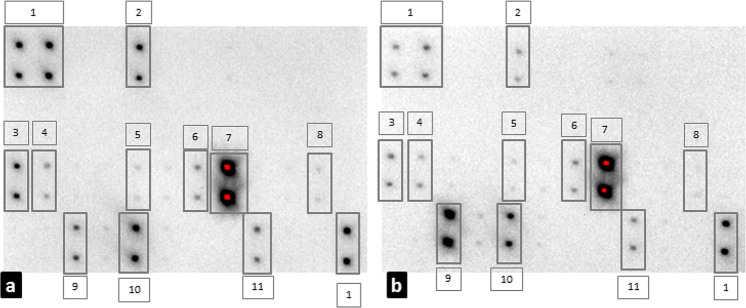
Cytokine array of a representative case of PAH (**a**) and a case of representative health control (**b**). The serum positivity was identified by the presence of a spot on the membrane. Figures a and b show a positive detection for (1) control, (2) ENA-78, (3) MCP-1, (4) MCP-2, (5) MDC, (6) MIP, (7) RANTES, (8) TARC, (9) EGF, (10) angiogenin, and (11) PDGF-bb

Among the 42 cytokines investigated in this study (Fig. 2), ANG, EGF, ENA78, GRO, MCP1, MCP2, MDC, MIG, MIP1, PDGF-bb, RANTES, TARC were identified in all PAH cases. No differences were identified between patients and controls at qualitative and quantitative analysis (Table 2). In fact, ANG, EGF, ENA78, GRO, MCP1, MCP2, MDC, MIG, MIP1, PDGF-bb, RANTES, TARC were detected also in health controls (Fig. 2).

**Table 2:** Univariate analysis of the distribution of the cytokines/chemokines signal intensity in cases and in controls

	Cytokines/chemokines quantitative analysis (signal intensity)		
	*PAH cases*	*Control*	*p value*
ANGIOGENIN	220306 (205462)	284031 (70280)	0.184
egf	452851 (286955)	873638 (115046)	0.07
ena78	128986 (83156)	99301 (12421)	0.685
gro	14876 (12774)	51315 (428)	0.003
mcp1	234138 (85871)	332482 (8837)	0.188
mcp2	63718 (22999)	40498 (37734)	0.327
mdc	29238 (14538)	46445 (22594)	0.267
mip1	99132 (39273)	81240 (52174)	0.621
PDGF-bb	101752 (30971)	142404 (21716)	0.07
rantes	1047955 (274300)	1190109 (210513)	0.409
tarc	28928 (14167)	48078 (3544)	0.075

### Cell-mediated immunity

In PAH patients, the mean values of neutrophils was 4.44 × 10^9/L (SD: 1.22, normal values: 1.9–7.0), of lymphocytes was 2.24 × 10^9/L (SD: 1.8, normal values: 0.9–5.2) and of the neutrophil/lymphocyte ratio was 2.1 (SD: 1.22, normal values: 1.4–2.1). The number of immune cells did not correlate with the presence or absence of APAs and AHAs, with the subtype of hypophysitis and with the occurrence of pituitary dysfunction, as shown in Table 3.

**Table 3:** Neutrophil count, the lymphocyte count and the neutrophil/lymphocyte ratio according to autoimmune pattern, hypophysitis subtypes and pituitary function. Univariate analysis

	Neutrophil count	*p* value	Lymphocyte count	*p* value	Neutrophil/ lymphocyte ratio	*p* value
Gender						
*Females n, (%)*	4.1 (1.7)	0.417	2.1 (0.6)	0.331	2 (1.2)	0.369
*Males n, (%)*	3.7 (0.9)		2.6 (0.9)		1.5 (0.7)	
APA						
Positivity	4.3 (1.6)	0.689	2.1 (0.6)	0.446	2.2 (1.4)	0.722
Negativity	4.7 (0.8)		2.4 (0.2)		1.9 (0.1)	
AHA						
Positivity	1.9 (1.7)	0.349	2.2 (0.7)	0.9	2.5 (1.8)	0.32
Negativity	4.1 (1)		2.3 (0.7)		2.8 (0.4)	
Hypophysitis subtypes						
AH	4.9 (1.7)	0.591	2.3 (0.6)	0.993	2.5 (1.8)	0.69
PH	4.3 (0.7)		2.3 (0.8)		1.9 (0.4)	
INH	3.9 (1.3)		2.2 (0.4)		1.8 (0.5)	
Secondary hypothyroidism						
Yes *n*, (%)	3.9 (0.4)	0.229	2.1 (0.9)	0.828	1.8 (0.7)	0.351
No *n*, (%)	4.05 (1.5)		2.2 (0.8)		2 (1.1)	
Secondary hypogonadism						
Yes *n*, (%)	4.3 (1.3)	0.601	2.5 (0.3)	0.359	1.7 (0.4)	0.617
No *n*, (%)	3.8 (1.6)		2.1 (0.8)		2 (1.3)	
Secondary hypoadrenalism						
Yes *n*, (%)	1.9 (0.5)	0.943	2.2 (0.6)	0.926	1.8 (0.5)	0.846
No *n*, (%)	3.9 (1.8)		2.2 (0.8)		1.9 (1.3)	
Growth hormone deficit						
Yes *n*, (%)	1.9 (1.5)	0.925	2.5 (0.2)	0.585	1.5 (0.7)	0.641
No *n*, (%)	4 (2)		2.2 (0.8)		2 (0.7)	
Hyperprolactinemia						
Yes *n*, (%)	3 (1.6)	0.275	1.4 (0.3)	0.03	2 (0.2)	0.827
No *n*, (%)	4.1 (1.6)		2.4 (0.7)		1.9 (1.3)	
Diabetes insipidus						
Yes *n*, (%)	3.7 (1)	0.641	2.3 (0.8)	0.571	1.7 (0.5)	0.421
No *n*, (%)	4.1 (2)		2 (0.7)		2.2 (1.6)	

## Discussion

PAH is recognized as an autoimmune disorder. The pathogenesis and the natural history of this disease were not completely clarified, although several studies were conducted to identify the putative antigens of PAHs, the HLA genotypes, the cell-mediated immune response [5–14]. In this study, we tried to investigate the immune response, though the determination of serum APAs and AHAs, serum cytokines and cell-mediated immunity.

APAs and AHAs are considered as markers of disease and, at the actual moment, their etio-pathogenetic role was not completely clarified [26], despite largely investigated [27]. Similarly, the clinical application of APAs and AHAs in the routine diagnosis is still debated for several methodological issues [28]. The indirect immunofluorescence is actually one of the more widely employed methods for detecting APAs and AHAs. However, same concerns still persist on the methodology, as the pituitary sections, that are usually used as substrates, were obtained from a variety of species and under different conditions [28]. APAs were detected in cryostat sections of pituitary glands obtained from humans, primates (monkey and young baboon), dogs and rodents [28, 29]. According to some authors, animal substrates have a lower sensitivity and specificity, as compared to human ones [30, 31]. Instead, other authors obtained superimposable or quite more reliable results using animal substrates as compared to human ones [32–34]. On 2014, Ricciuti et al. reviewed systematically the articles that investigated the APA by indirect immunofluorescence, suggesting that human pituitary is the most suitable tissue for the detection of APAs, in particular if treated with Sudan black B, in order to reduce the pituitary autofluorescence [29]. According to these authors, the cytosolic APA staining turned out to be the best indicator of an autoimmune pituitary pathology and is therefore a finding that can be useful to clinicians in establishing a diagnosis of hypophysitis [29]. However, although we applied a different immunofluorescence technique, our results are in line with those of Ricciuti et al. [29]. In fact, in our series, APAs were detected in around 68% of hypophysitis cases and in Ricciuti et al. series in 40.9% of cases, resulting in both the studies more frequently than in health controls. In particular, in our series, APAs were identified in 0.5% of health controls, 6% of cases of primary empty sella [35], 12% of NFPAs, 10% of GH-secreting pituitary adenomas and 8% of prolactin-secreting pituitary adenomas [25].

In this study, the positivity for APA and of AHA was detected in 68.4% of cases. All patients had at least a positivity for AHA or APA and the coexisting detection of APA and AHA were detected in 10 patients (52.9%). In this study, the prevalence of APA and AHA was significantly higher in PAHs, in comparison to health controls and NFPAs. We found that the detection of APA and AHA was protective from the diagnosis of NFPA, in particular in cases with a simultaneous positivity of APAs and AHAs. As for consequence, the detection of APAs and AHAs may be useful for the differential diagnosis between PAHs and NFPAs. This report underlines the importance of testing APAs and AHAs in the correct clinical context, as patients with hypopituitarism and/or neurological/ophthalmological signs of sellar/parasellar mass and with the typical radiological sign of hypophysitis and after the exclusion of focal pituitary lesions [16, 19].

A limitation of our study is the absence of a comparative analysis of prevalence of APAs with different immunofluorescence substrates. However, our reports confirmed previous studies [29], showing that APAs can discriminated between patients affected by hypophysitis and health controls.

At the actual moment, the immunofluorescence method is still discussed [28], as data on sensitivity and specificity of the human or animal pituitary substrates for the research of APAs are not conclusive. In addition, pituitaries of primates remain the most used substrate for the detection of APA, as human pituitary is extremely difficult to source and commercial kit are not available [28]. Another limitation of this study is the absence of data on the pattern of cytosolic staining. In this study, as suggested also by other authors [20, 21], the samples were considered positive when a diffuse immunofluorescence pattern with an intracytoplasmic staining was observed in most fields. However, same author suggested also that a granular cytosolic staining was highly predictive of pituitary autoimmunity [29].

An interesting finding of this study is related to the evaluation of the cell-mediated immunity and serum cytokines. T lymphocytes are considered the main inducers of damage in organ-specific autoimmune diseases [26]. In fact, in a recent study conducted on a murine experimental model of PAH, Lin et al. demonstrated that CD3- and B220 lymphocytes proliferate within the pituitary and secrete gamma-interferon and interleukin-17. Our finding may confirm this hypothesis that pituitary-infiltrating lymphocytes proliferate “in-situ” [36], as we found that serum cytokines and white blood cells count were similar between PAH cases and health controls, suggesting that PAH may be a localized autoimmune disease, rather than a systemic one.

In conclusion this study suggests that APA and AHA may be detected in an high percentage of PAH cases and that their simultaneous identification may be useful for the differential diagnosis between PAH and NFPAs, in an appropriate clinical context.
